# Competition and constraint drove Cope's rule in the evolution of giant flying reptiles

**DOI:** 10.1038/ncomms4567

**Published:** 2014-04-02

**Authors:** Roger B. J. Benson, Rachel A. Frigot, Anjali Goswami, Brian Andres, Richard J. Butler

**Affiliations:** 1 Department of Earth Sciences, University of Oxford, Oxford OX1 3AN, UK; 2 Center for Functional Anatomy and Evolution, Johns Hopkins University, Baltimore, Maryland 21205, USA; 3 Department of Genetics, Evolution & Environment and Department of Earth Sciences, University College London, London WC1E 6BT, UK; 4 School of Geosciences, University of South Florida, Tampa, Florida 33620, USA; 5 School of Geography, Earth and Environmental Sciences, University of Birmingham, Birmingham B15 2TT, UK

## Abstract

The pterosaurs, Mesozoic flying reptiles, attained wingspans of more than 10 m that greatly exceed the largest birds and challenge our understanding of size limits in flying animals. Pterosaurs have been used to illustrate Cope’s rule, the influential generalization that evolutionary lineages trend to increasingly large body sizes. However, unambiguous examples of Cope’s rule operating on extended timescales in large clades remain elusive, and the phylogenetic pattern and possible drivers of pterosaur gigantism are uncertain. Here we show 70 million years of highly constrained early evolution, followed by almost 80 million years of sustained, multi-lineage body size increases in pterosaurs. These results are supported by maximum-likelihood modelling of a comprehensive new pterosaur data set. The transition between these macroevolutionary regimes is coincident with the Early Cretaceous adaptive radiation of birds, supporting controversial hypotheses of bird–pterosaur competition, and suggesting that evolutionary competition can act as a macroevolutionary driver on extended geological timescales.

## Abstract (web summary)

Pterosaurs were Mesozoic flying reptiles with extremely large body sizes. Here, Benson *et al.* demonstrate that pterosaurs evolved increasing body sizes during the Cretaceous, at the same time of bird radiation, suggesting that competition can drive macroevolution.

## Graphical abstract

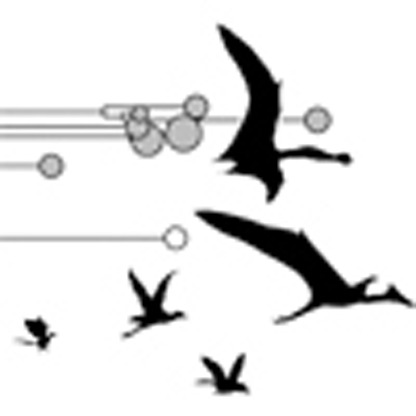

The pterosaurs, a diverse clade of Mesozoic flying reptiles, achieved body sizes unparalleled by other airborne organisms in Earth’s history^1^. Maximum wingspans over 10 m are twice those of the largest birds, exceed many small aircraft, and have fundamentally challenged our biomechanical understanding of the limits to body size in flying animals^2^,^3^. Marked increases in pterosaur body size through their >150 million year evolutionary history have lead to their frequent use as examples of Cope’s rule^4^, the influential hypothesis that active selection generates trends of increasing body size through time^5^,^6^. However, the generality of this ‘rule’, its ability to persist in large clades comprising numerous, independently evolving lineages over timescales of 10^7^–10^8^ years, and the existence of sustained, long-term drivers of such trends, are widely questioned^7^,^8^,^9^,^10^,^11^. Well-documented examples of Cope’s rule are restricted to specialized clades (for example, herbivores and hypercarnivores) within larger radiations such as mammals^5^ or carnivorans^6^. This indicates that adaptation to niches requiring large body size drives evolutionary change along individual lineages. However, it does not provide evidence that phylogenetically inclusive trends towards gigantism are common within major animal radiations.

Previous authors have suggested that Cope’s rule applied to some groups of pterosaurs, and identified temporal and taxonomic variation in pterosaur body size evolution^4^,^12^,^13^. However, the explanatory power of these studies was limited by: (i) small data sets; (ii) failure to use detailed phylogenetic information; and (iii) methods that do not reliably distinguish active selection for body size increases along evolutionary lineages from those driven by species sorting (for example, selective extinction of smaller taxa^14^,^15^). Because of this, the evolutionary processes underlying pterosaur gigantism remain elusive.

A classic, but controversial, hypothesis posits competitive replacement of pterosaurs by birds^16^,^17^,^18^. One version of this hypothesis suggests that early birds excluded pterosaurs from small-bodied flying niches, causing selective extinction of small-bodied pterosaurs^19^,^20^, and perhaps driving pterosaur lineages to evolve larger sizes^4^. However, quantitative studies of limb proportion morphospace occupation and species richness led recent authors to suggest that birds did not compete with pterosaurs^21^,^22^. Indeed, although competition between species might occur on microevolutionary timescales, clear examples of competition as a macroevolutionary driver are rare^23^,^24^.

We examined phylogenetic and non-phylogenetic patterns of pterosaur body size evolution using a large phylogeny and data set of wingspan measurements and estimates. Our results indicate that pterosaurs exhibit an extremely long term, whole-clade trend to increasing body sizes during the Cretaceous, coincident with the adaptive radiation of pygostylian birds. This suggests that competition between distantly related clades (for example, birds and pterosaurs) can act as a macroevolutionary driver on extended timescales.

## Results

### Pterosaur body size through time

**Figure 1:**
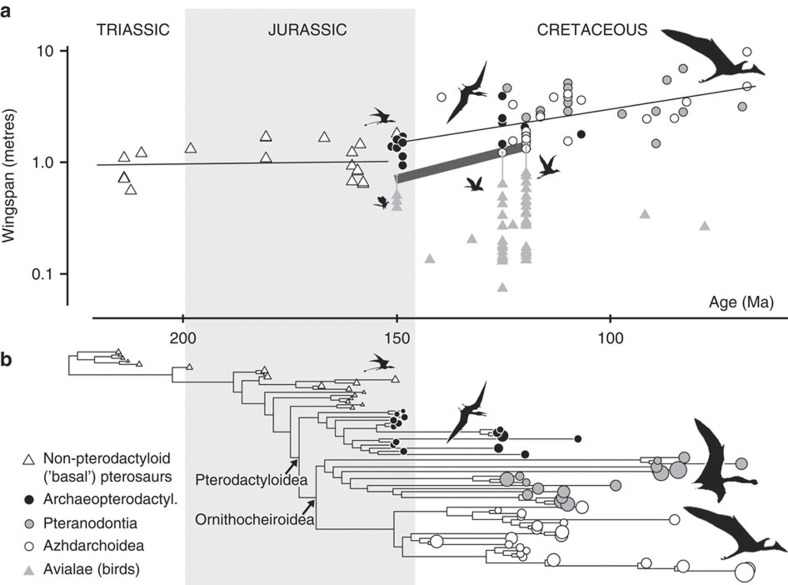
Pterosaur and bird size through time. (**a**) Pterosaur adult wingspan versus geological age in millions of years (Ma). Bird datapoints represent osteological wingspans, but the likely increment to maximum wingspans from flight feathers is indicated by grey whiskers (50% in *Archaeopteryx*; 100% in Early Cretaceous birds). The thick grey line indicates maximum bird wingspans during the well-sampled Late Jurassic–Early Cretaceous interval. Thin black lines are major axis regression lines of pterosaur wingspans on geological age. The confidence interval of the Triassic–Jurassic regression line includes zero, indicating an absence of size increases (*P*=0.396 (regression slope); *N=*18; slope= −0.0004 (−0.0040 to 0.0031) log_10_(m)/Ma; intercept=0.079 log_10_(m) (=1.20 m)); the regression line for the latest Jurassic–Cretaceous indicates increasing body size through time (*P*<0.001 (regression slope); *N=*54; slope= −0.0060 (−0.0082 to −0.0039) log_10_(m)/Ma). (**b**) One representative time-calibrated cladogram of adult pterosaur specimens with tip diameters proportional to log_10_(wingspan). In both **a**,**b**, silhouettes are indicative of relative size only.

For the first 70 million years of their evolutionary history, during the Triassic and Jurassic, pterosaur body sizes showed stable variance about a sub-constant mean, suggesting highly constrained body size evolution in basal (non-pterodactyloid) pterosaurs (Fig. 1a). Sustained increases in maximum and minimum body size began between 150 and 130 Ma, continuing for 65–85 million years, until pterosaur extinction at the Cretaceous–Palaeogene boundary. During this second interval, body sizes show no evidence of increased variance through time (on a logarithmic scale; Fig. 1a), and so are consistent with a driven evolutionary trend^25^. Our results demonstrate an abrupt upwards inflection in pterosaur wingspans some time between the latest Jurassic and Early Cretaceous, around the time of the appearance and subsequent adaptive radiation of birds^26^,^27^.

### Macroevolutionary models

**Figure 2:**
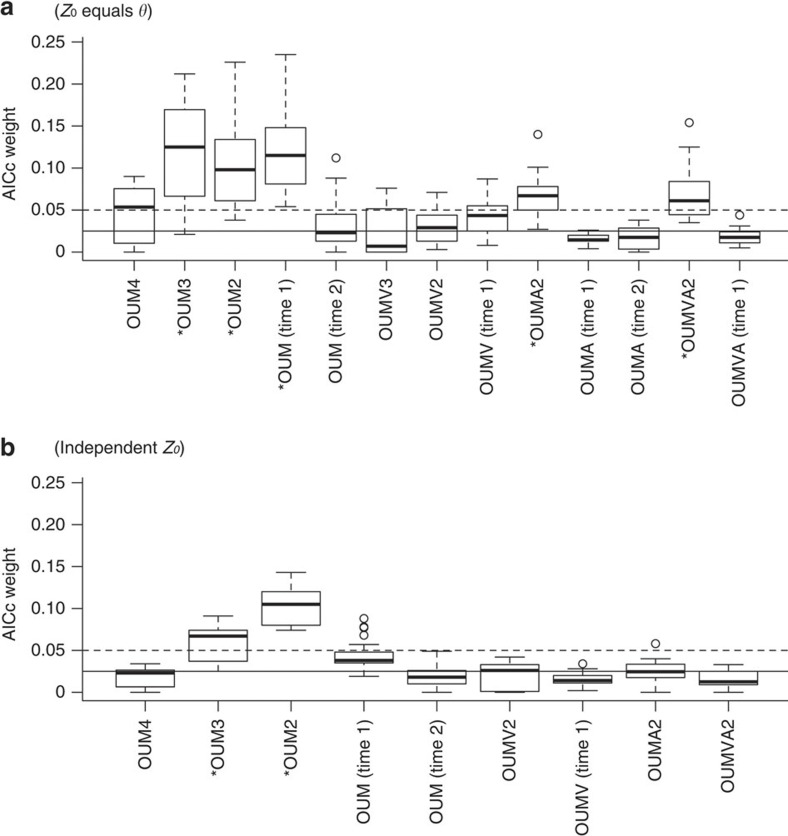
Model support for pterosaur body size evolution. AICc weights are shown for (**a**) models in which the root node value (*Z*_0_) is equal to the macroevolutionary optimum for basal (non-pterodactyloid) pterosaurs (*θ*). (**b**) Models in which the root node value is estimated separately from the macroevolutionary optimum for basal pterosaurs. *indicates models with median AICc weights above an arbitrarily-specified value of 0.05. Results from the following models are shown: OUM is an Ornstein–Uhlenbeck model with group-specific trait optima (*θ*); OUMV allows a group-specific *θ* and stochastic rate parameter (*β*, Brownian variance); OUMA allows group-specific *θ* and attraction parameters (*α*). Model name suffixes indicate the number of groups specified with distinct macroevolutionary regimes: 2, basal (non-pterodactyloids) and Pterodactyloidea; 3, basal, Archaeopterodactyloidea and Ornithocheiroidea; 4, basal, Archaeopterodactyloidea, Pteranodontoidea and Azhdarchoidea; time 1, Triassic–Kimmeridgian and Tithonian–Cretaceous; time 2, Triassic–Tithonian and Cretaceous. Thick lines indicate median values across 25 time-calibrated phylogenies, boxes indicate interquartile ranges, whiskers indicate ranges excluding outliers, and circles indicate outliers. Dashed line occurs at 0.05, non-dashed, horizontal line occurs at 0.025.

All of the best phylogenetic models suggest that pterodactyloids had larger optimal body sizes than non-pterodactyloids, and some suggest of them larger optimal sizes in ornithocheiroid pterodactyloids compared with non-ornithocheiroid pterodactyloids. AICc (Akaike’s Information Criterion for finite sample sizes) weights for these clade-specific alternatives are comparable to those of a temporal regime shift occurring late in the Jurassic (Tithonian), around the origination date of birds (Fig. 2 and Table 1).

Some of the best models suggest a macroevolutionary optimum (*θ*) for non-pterodactyloids (*N=*19) that is different to the root node value (*Z*_0_) (Table 1: models D and F, both taking values ~1 m wingspan). However, AICc weights do not distinguish this from the possibility that the non-pterodactyloid optimum is equal to the root node optimization. Critically, estimation errors for both root node and non-pterodactyloid optimum values are high enough that their confidence intervals overlap (Table 1). The possibility that non-pterodactyloid optimum and root node values are approximately equal is consistent with stasis-like, constraint-dominated body size evolution in non-pterodactyloids^28^,^29^,^30^.

Broadly two classes of solution, which form a continuum, are recovered for pterodactyloid body size optima:

In one class, macroevolutionary optima for pterodactyloids as a whole (3.81 m; 0.581 log_10_m; *N=*53), Tithonian and younger pterosaurs (including some non-pterodactyloids; 4.01 m; 0.603 log_10_m; *N=*52), or archaeopterodactyloids (2.46 m; 0.391 log_10_m; *N=*15) and ornithocheiroids (4.16 m; 0.619 log_10_m; *N=*38) individually, fall within their observed range of wingspans, and attraction to optima is relatively strong (*α*=0.0067–0.0282; Table 1: models A–C). This suggests that pterodactyloids were attracted to larger body size optima than non-pterodactyloids through the Cretaceous, and also that temporally successive clades might have had progressively larger body size optima.

In the other class of solutions, macroevolutionary optima of pterodactyloid body size commonly exceed the range of observed values (Table 1: models D–G), but attraction to optima (*α*) is weak in pterosaurs (*α*=0.0011–0.0210), and might be weaker in pterodactyloids than in non-pterodactyloids (Table 1: models E and G). Models with large optima, but low attraction, indicate a sustained directional trend towards larger size, because as *α* converges on zero, the Ornstein–Uhlenbeck (OU) model reduces to Brownian motion (BM) with a constant trend coefficient *μ* (ref. 28), estimated ~0.0050–0.0100 log_10_(m)/Ma (Table 1: model D; median results for ±2 s.e. *θ*, using *μ*=*α* × *θ*; (ref. 28), p. 1348) or 0.0078–0.0133 (Table 1: model E; a worse model). The value of this coefficient is similar to the absolute slope of the non-phylogenetic regression line fit to Cretaceous pterosaur body size (0.0060 log_10_(m)/Ma), and is within (or overlaps with) its 95% confidence intervals: 0.0039–0.0082 log_10_(m)/Ma. This suggests that all, or almost all, of the observed pattern of Cretaceous pterosaur body size increase can be explained by directional evolution along lineages. Therefore, if this model is correct, then among-lineage factors such as selective extinction had a negligible role in determining pterosaur body size distributions at the resolution of our study^14^.

## Discussion

Pterosaur body sizes showed sustained evolutionary increases for the last 65–85 million years of their existence, culminating in giant flying animals with wingspans of 3–10 metres. This might have been achieved by sustained directional evolution along individual lineages, or by increasingly large macroevolutionary optima in temporally successive clades. Whichever is the case, the documented pattern represents a striking example of long-term directional trend in the fossil record. The result is all the more conspicuous because earlier pterosaur body size evolution was constrained to a very limited range of relatively small adult body sizes (<1.6 m) and showed no evidence of directional change through time.

This fundamental shift in the mode of body size evolution across Pterosauria suggests a long-term, phylogenetically inclusive driver. We recognize the following two major hypotheses that are not mutually exclusive^19^:

(1) Intrinsic features of the pterodactyloid body plan, such as modifications of the tail and limbs, which allowed improved terrestrial abilities and diversification of flight styles facilitated invasion of new, large-bodied niches^3^,^19^,^31^.

(2) Extrinsic factors such as the Late Jurassic appearance and Cretaceous diversification of birds^26^,^27^ may have excluded pterosaurs from the ecological niches available to smaller flying animals, causing selective extinction of smaller pterosaurs, and also possibly causing active selection for larger body size in pterosaur evolution.

Intrinsic factors explain how pterodactyloids were able to exceed previous constraints on basal pterosaur body size, and continuously extend their maximum body size throughout the Cretaceous. However, intrinsic factors acting alone predict expansion into a greater range of body sizes and do not explain the loss of smaller-sized pterosaurs^14^,^25^. Extrinsic forcing must therefore also have been important. Indeed, directional models of along-lineage evolution are sufficient to explain all of the observed increase in Cretaceous pterodactyloid body size at the scale of this study (for example, Table 1: model D). However, these models receive approximately equal support to models of successively higher optima in temporally younger clades (for example, Table 1: model A), which allow a partial role for selective extinction in explaining pterosaur body size trends.

Birds (Avialae) originated in the Late Jurassic, and key aspects of the avian body plan, which enabled the ecological and taxonomic diversification of birds, occurred from the Early Cretaceous^26^,^27^. Forcing by a sustained, Cretaceous adaptive radiation of birds could explain how increases in pterodactyloid minimum body size were sustained over a remarkably long-time interval, and why most or all smaller pterosaurs became abruptly extinct around the Jurassic–Cretaceous boundary (non-pterodactyloids). Although the small pterosaur *Dendrorhynchoides* was originally reported from the Cretaceous of China^32^, its provenance is uncertain and it might be of Jurassic age^33^.

Birds underwent expansion into both smaller and larger body sizes during the Early Cretaceous (Fig. 1a). Their pattern of increasing body size variance suggests an unconstrained or ‘passive’ pattern of body size diversification^25^, unlike the active, directional pattern seen in pterosaurs of the same time interval. Strikingly, the maximum skeletal wingspans of birds appear to increase at approximately the same rate as minimum pterosaur wingspans during the Late Jurassic–Early Cretaceous (Fig. 1a). The rarity of bird skeletons complete enough for wingspan estimation means that this pattern cannot yet be assessed in the Late Cretaceous. However, fragmentary remains suggest body masses of 3–5 kg for some likely volant latest Cretaceous birds, which are among the largest of the Mesozoic^34^ and demonstrate persistent increases in maximum body size. Furthermore, it is clear that Late Cretaceous birds occupied an ever increasing number of those ecological niches available to smaller or medium-sized flying animals, and continued to diversify in body size and shape^26^,^27^, raising the possibility that they exerted competitive pressure on an expanding range of ecological niches. Early Cretaceous pterosaurs took advantage of new large body-sized niches, and might have had comparable diversity and disparity to Jurassic pterosaurs^35^,^36^,^37^. However, it is clear that pterosaur species diversity^35^ and morphological disparity^36^,^37^ declined during the Late Cretaceous until their final extinction, coinciding with the culmination of their pattern of escalating body size.

When estimated feather lengths are included, the largest birds have similar wingspans to smaller contemporaneous pterosaurs in well-sampled Cretaceous time intervals. This does not appear to have been the case during the latest Jurassic (Fig. 1a). However, some latest Jurassic pterosaurs are known from subadult individuals that might have had small adult wingspans (for example, the non-pterodactyloid *Anurognathus*) but could not be included in our analyses. If smaller latest Jurassic pterosaurs existed, then the pattern of increasing pterosaur wingspans might have begun later than our results suggest, during the Early Cretaceous instead of the latest Jurassic. This possibility is consistent with the lack of ecological and taxonomic diversity among latest Jurassic birds (a single genus, *Archaeopteryx*). Competitive interactions occur on generational timescales, which cannot usually be resolved in the fossil record^38^. Therefore, the apparent temporal delay between the disappearance of small, Late Jurassic pterosaurs and the appearance of ecologically diverse Early Cretaceous birds with comparable wingspans to those pterosaurs is not evidence of non-competition. Instead, it might reflect the poor earliest Cretaceous record of both birds and pterosaurs (Fig. 1).

Our arguments generally rest on observations of similar body sizes in contemporaneous birds and pterosaurs. However, smaller birds might also have had a significant role in competing with pterosaurs. Pterosaurs and birds are separated from one another by substantial phylogenetic distance and might have had distinct energetic requirements, life history strategies and functional means of exploiting similar ecological niches. In these circumstances, strong competitive interactions can exist between animals of different sizes in modern ecosystems, including examples in which smaller taxa have the competitive advantage (for example, the invasive cyprinid fish *Pseudorasbora parva*^39^).

Several observations contribute to the plausibility of competitive ecological interactions between pterosaurs and birds:

(1) Both clades co-occur in the same marginal marine and continental units during the Late Jurassic^40^ and Cretaceous^41^, demonstrating overlap in environmental and geographic distributions^38^. Interestingly, birds are substantially more abundant and diverse than pterosaurs in inland environments from the Early Cretaceous^42^, whereas pterosaurs remained abundant in coastal environments^43^. However, the Late Cretaceous decline of pterosaurs, even those living in coastal environments^35^,^36^,^37^, is coincident with a Late Cretaceous diversification of coastal birds^26^.

(2) Similar diets are hypothesized for many pterosaurs and birds from Early Cretaceous units, including fish, seeds, invertebrates and hard food items in the Jehol Biota and other faunas^26^,^44^. Critically, many pterodactyloids, including most non-pterodactyloids, are characterized as eating fish^19^, which are included in the fossilized gut contents of Early Cretaceous birds^45^. These observations all demonstrate consumption of common resources by many birds and pterosaurs^38^. Future discoveries of Late Cretaceous birds will provide a further test of evolutionary interactions between birds and pterosaurs.

Our interpretation of pterosaur body size evolution resulting from bird diversification differs from those of previous studies asserting a lack of ecological interactions between birds and pterosaurs. For example, McGowan and Dyke^21^ suggested that differences in the limb proportion of birds and pterosaurs demonstrated that they occupied different niches. Butler *et al.*^22^ suggested that pterosaur diversity did not decline until the Late Cretaceous, and proposed that it was difficult to attribute these late diversity losses to competition with birds. However, given the pronounced structural differences between bird and pterosaur wings, it is not clear that differences in their limb proportions indicate substantial differences in ecological niche occupation rather than differences in flight mechanics. Furthermore, Early Cretaceous losses of small-bodied pterosaur species due to competition with birds might have been balanced by gains in pterosaur diversity at large body sizes, or by pterodactyloids exploiting new ecological niches, especially in coastal environments^19^. This could have delayed the apparent decline of overall pterosaur diversity.

Pterosaurs became extinct at the end of the Cretaceous, at a time when their wingspans ranged from 3 to 10 m. Whether pterosaurs would have survived the Cretaceous–Palaeogene extinction if they had retained their earlier, smaller body size niches into the latest Cretaceous cannot be known. However, exponentially fewer niches are available for larger animals^46^, a well-known correlation between increasing body size and extinction risk exists for modern birds^47^ and other groups^48^, and large terrestrial animals were disproportionately affected by the Cretaceous–Palaeogene extinction^49^,^50^. It seems likely that the absence of such small, late-surviving pterosaurs was a result of the ecological radiation of early birds. If so, competition with birds was responsible in part for the ultimate demise of these spectacular animals.

## Methods

All analyses were performed in R version 3.0.0 (ref. 51) using log_10_-transformed data. Our data set and code are archived at DRYAD ( doi:10.5061/dryad.n0310).

### Data set

We assembled the following data on the preserved skull and forelimb elements in 168 nominal pterosaur species: skull length, mandible length, preorbital rostrum length, humerus length, ulna length excluding olecranon process, radius length, metacarpal IV length and the lengths of phalanges 1–4 of the wing finger (digit IV) (Supplementary Data 1). Data was collected via direct specimen measurement by B.A. and from literature sources. For 34 taxa with complete forelimbs, wingspans were calculated as the summed lengths of the measured forelimb elements. Wingspans were log_10_-transformed before analysis because relative, not absolute, changes form the natural scale of phenotypic evolution^46^. Pterosaur phylogeny was based on a new taxon-rich and well-resolved cladogram including 109 species^52^, 92 of which had wingspans that could be measured or estimated (Fig. 1b), and temporally calibrated to geological age using paleotree version 1.8.2 (ref. 53).

The geological ages of most taxa were resolved to stage or substage level. These were converted to numerical maximum and minimum ages using the timescale of Gradstein *et al.*^54^

We also collected comparable osteological wingspan data for 50 Mesozoic birds^27^ (Supplementary Data 2) so that pterosaur body size and avialan body size during the Cretaceous radiation of birds could be compared. Pterosaur and bird wings are constructed differently. In pterosaurs, the forelimb skeleton spans the entire length of the membranous wing. In contrast, bird wings include a large non-osteological portion comprised by flight feathers. Only a few Mesozoic bird fossils preserve feathers sufficiently complete to measure their proportional contribution to estimated wingspan. However, among these taxa, feathers add 50% in *Archaeopteryx*, or ~100% in Cretaceous birds, to the summed length of the forelimb bones (Supplementary Data 2). The effects of this are indicated in Fig. 1.

### Time-calibrated phylogenies

Phylogenetic approaches were based on the strict consensus cladogram reported by Andres and Myers^52^ that includes 109 taxa and 105 resolved nodes. The remaining nodes were resolved randomly and alternative randomizations had little effect on our results. Tip ages (that is, the ages of taxa in the tree) were drawn randomly from a uniform distribution between the maximum and minimum possible ages of each taxon. These were used to generate minimum node ages. The resulting zero-length branches of our cladogram were extended by imposing a minimum branch length of 2 Ma. All randomizations were implemented using the timePaleoPhy function of the R package paleotree version 1.8.2 (ref. 53), and analyses carried out on different versions of the time-calibrated tree yielded similar results to those reported here. We also calibrated trees using a minimum branch length of 1 Ma, and using the ‘equal’ method, which extends zero-length branches by sharing duration from the more basal non-zero-length branches. Analyses on these phylogenies yielded similar results to those reported.

### Missing data estimation—phalanx IV-4

Twelve taxa with incomplete forelimbs were missing only the terminal wing finger phalanx (phalanx IV-4: phalanx 4 of digit IV). Therefore, we estimated the missing phalanx IV-4 lengths, resulting in a total of 47 wingspans that could be calculated directly. To determine the best estimation formula, we compared phylogenetic and non-phylogenetic generalized least squares regression models^55^,^56^ using the R packages ape version 3.0–8 (ref. 57) and nlme version 3.1–109 (ref. 58) to establish which other element of the skull or forelimb best predicted the length of phalanx IV-4. We used AICc^59^,^60^ to determine the level of phylogenetic signal in each regression relationship by comparing models with three different values of the phylogenetic signal parameter lambda *λ* (ref. 61): (1) *λ*=0, equivalent to ordinary least squares regression (non-phylogenetic); (2) *λ*=1, equivalent to regression phylogenetic independent contrasts; and (3) *λ* allowed to vary, and estimated using maximum-likelihood.

For multiple phylogenies (see below), the best predictive regression model for the length of phalanx IV-4 is phylogenetic regression (*λ*=1) on the length of phalanx IV-3, with an *R*^2^=0.86. When *λ* is allowed to vary and estimated separately, a value of 0.96 is obtained (approximately equal to 1.0). This result supports the use of phylogenetically informed estimation methods^62^ based on phylogenetic regression of phalanx IV-4 length on phalanx IV-3 length to predict the length of phalanx IV-4. In confirmation of this approach, the predicted values of taxa for which the length of phalanx IV-4 is known are strongly correlated with real values (*R*^2^=0.85) and become substantially stronger when *Jeholopterus*, which has an atypically short phalanx IV-4 (ref. 63), is removed from comparisons (*R*^2^=0.92). Once this is done, the mean absolute difference between predicted and known measurements is 0.058 log_10_mm, which is negligible, compared with the mean (1.74 log_10_mm) and s.d. (0.25 log_10_mm) of its absolute length across our taxon sample.

### Missing data estimation—wingspan

To establish the best regression model for estimation of unknown wingspans, we used the protocol applied above a second time. The results are shown in Table 2, excluding *Jeholopterus*, which has an unusually short skull and phalanx IV-4 (ref. 63), and is therefore an outlier in several analyses. For regression of wingspan on skull length, mandible length, rostrum length, humerus length, metacarpal IV length and the lengths of phalanges IV-1, IV-2, IV-3 and IV-4, phylogenetic regression provides the best model, and independently estimated *λ* values are approximately equal to 1.0 (range: 0.90–1.10). However, for regression of wingspan on radius or ulna length, non-phylogenetic regression provides the best model, and estimated *λ* values are approximately equal to zero (Table 2). The absence of phylogenetic signal in the relationship between wingspan and ulna/radius length implies a strong functional or developmental correlation between these measurements.

Based on *R*^2^ values, it is clear that phalanges IV-1 and IV-2 provide the best (phylogenetic) predictions of pterosaur wingspan, and ulna length might also provide good (non-phylogenetic) predictions (Table 2). But these variables only enable a few additional wingspan estimates (12–19 estimates; Table 2). Humerus length allows more additional estimates (27 estimates) but apparently has a slightly weaker relationship with wingspan (Table 2). Mandible length is the best cranial predictor of pterosaur wingspan. When known wingspans are estimated using these relationships, the mean standard errors are low (Table 2), and favour use of phalanx lengths, humerus length and mandible length to maximize the number and accuracy of estimates for pterosaurs in which wingspan was not known. In our analyses, we used wingspans from directly measured values preferentially. Where these were absent, we preferred estimated wingspans based on the length of phalanx II-4, then phalanx I-4, then the humerus and then the mandible. This resulted in 72 wingspans or wingspan estimates associated with estimates of standard error.

Because we used the skeletal proportions of specimens to estimate pterosaur wingspans, our data collection focussed only on complete individuals and not on maximum-sized or adult individuals. Therefore, our wingspans were known to be underestimates in several taxa. The wingspans of the following taxa were scaled up by the proportions of their largest individual (Supplementary Data 1): *Ardeadactylus longicollum*, *Batrachognathus volans*, *Campylognathoides liasicus*, *Darwinopterus modularis*, *Dimorpodon macronyx*, *Dorygnathus banthensis*, *Eudimorphodon rosenfeldi*, *Germanodactylus rhamphastinus*, *Noripterus parvus*, *Pteranodon longiceps*, *Pterodactylus antiquus*, *Pterodaustro guinazui*, *Rhamphorhynchus muensteri*, *Sinopterus dongi* and *Zhejiangopterus linhaiensis*.

### Pterosaur body size through time

In addition to phylogenetic model fitting (explained below), we also fit (non-phylogenetic) ordinary least squares regression lines to wingspan versus age separately for Triassic–Jurassic and Cretaceous pterosaurs. These lines capture the long-term trajectory of pterosaur body size distributions that emerges from interactions between along-lineage evolution (models described above) and among-lineage factors such as species sorting^14^.

### Macroevolutionary models

Analyses were conducted excluding taxa known only from juveniles and subadults, identified using well-established skeletal ossification and fusion characters^64^,^65^). In total, 72 adult wingspans, or estimated wingspans with mean standard errors, were known for taxa included in our tree.

BM is a two parameter model in which *β* is the Brownian variance, a measure of evolutionary rate^29^,^66^, and *Z*_0_ is the root node value. OU is a three/four parameter model of evolution constrained around a trait optimum (*θ*) by strength of attraction *α*^28^,^67^,^68^. In the OU model, *β* becomes the dominant variable when trait values are approximately equal *θ*. When this occurs, *β* specifies expected trait variance about a mean value of *θ*, thus mimicking macroevolutionary stasis^28^,^29^. The trait optimum of OU models can be set equal to the root node optimization (*Z*_0_), or for non-ultrametric trees a separate value can be estimated (*θ*), resulting in a four parameter model of attraction towards the trait optimum from a distinct root node value^67^.

Hypotheses of non-Brownian body size evolution on our time-calibrated trees were examined by testing the fit of numerical evolutionary models using maximum likelihood^15^,^61^,^66^,^67^,^68^, using the R packages geiger version 1.99–3 (ref. 69) for exploratory analyses, and OUwie version 1.33 (ref. 67) for the full set of final analyses. Although geiger includes some models that cannot be tested in OUwie, most of these (early burst and the ‘white’ stasis model) had poor fit to our data compared with BM or the OU model^28^,^67^, and trend (or ‘drift’) can effectively be modelled by an OU model (ref. 28, p. 1348).

Alternative macroevolutionary models were evaluated in Pterosauria and subsets of Pterosauria including non-pterodactyloid grade pterosaurs, Pterodactyloidea, the two major pterodactyloid clades Archaeopterodactyloidea and Ornithocheiroidea, the ornithocheiroid clades Pteranodontoidea and Azhdarchoidea, and between time intervals specified using the make.era.map function of phytools^70^. Models were compared using Akaike weights^60^ based on AICc^59^.

Single regime models (that is, one set of evolutionary model parameters applied to the whole tree) were compared with multiple regime models in which stochastic rates (*β*), body size optima (*θ*) and strength of attraction (*α*) to optima vary between groups^67^. Specifically, we tested whether modes of evolution varied among non-pterodactyloid pterosaurs, pterodactyloids and pterodactyloid subclades (Archaeopterodactyloidea, Ornithocheiroidea, Pteranodontoidea and Azhdarchoidea), and between two pairs of time intervals: (1) Triassic–Kimmeridgian and Tithonian–Cretaceous; and (2) Triassic–Tithonian and Cretaceous; note that the Tithonian stage of the latest Jurassic marks the first fossil occurrence of Avialae: *Archaeopteryx*).

Owing to the large numbers of parameters, and the explicit inclusion of estimation errors and stratigraphic uncertainty in our data, model optimization was not successful for all analyses. For example, sometimes, probable local optima or saddle points in the likelihood surface were recovered. The results reported here are those from 25 time-calibrated phylogenies with successfully optimized parameters across all the candidate models. These results are representative of those recovered in other analyses.

## Author contributions

R.B.J.B., A.G. and R.J.B. designed the research; and R.B.J.B. performed the analyses and assembled the bird data set. All authors contributed to the pterosaur data set and to writing the manuscript.

## Additional information

**How to cite this article:** Benson, R. B. J. *et al.* Competition and constraint drove Cope's rule in the evolution of giant flying reptiles. *Nat. Commun.* 5:3567 doi: 10.1038/ncomms4567 (2014).

## Supplementary Material

Supplementary Data 1 Pterosaur measurements, geological ages, and wingspans

Supplementary Data 2 Mesozoic bird measurements

## Figures and Tables

**Table 1:** Macroevolutionary model parameters for pterosaur body size evolution.

	**Model**	**N**_**groups**_	**AICc weight**	**Group/interval**	**Attraction (*****α*****) and phylogenetic half-life (in square brackets)**		**Stochastic rate (*****β*****)**		**Optimum (*****θ*****) or root value (*****Z***_**0**_**)**		**s.e. of** ***θ*** **or** ***Z***_**0**_	
			**Median**		**Median**	**Range**	**Median**	**Range**	**Median**	**Range**	**Median**	**Range**
A	OUM	3	0.125	Basal	0.0158 [48.9]	(0.0106–0.0195)	0.00078	(0.00060–0.00093)	**−0.001**	**(−0.019 to −0.007)**	0.076	(0.072–0.079)
				Archaeopterodactyloidea	0.0158 [48.9]	(0.0106–0.0195)	0.00078	(0.00060–0.00093)	**0.391**	**(0.318–0.475)**	0.155	(0.136–0.181)
				Ornithocheiroidea	0.0158 [48.9]	(0.0106–0.0195)	0.00078	(0.00060–0.00093)	**0.619**	**(0.552–0.713)**	0.092	(0.085–0.106)
B	OUM	2	0.115	Triassic–Kimmeridgian	0.0206 [33.6]	(0.0150–0.0282)	0.00094	(0.00068–0.00123)	**0.000**	**(−0.012–0.005)**	0.076	(0.073–0.079)
				Tithonian–Cretaceous	0.0206 [33.6]	(0.0150–0.0282)	0.00094	(0.00068–0.00123)	**0.603**	**(0.527–0.684)**	0.069	(0.058–0.086)
C	OUM	2	0.105	Basal	0.0106 [65.4]	(0.0067–0.0148)	0.00077	(0.00061–0.00089)	**−0.016**	**(−0.026 to −0.013)**	0.080	(0.074–0.082)
				Pterodactyloidea	0.0106 [65.4]	(0.0067–0.0148)	0.00077	(0.00061–0.00089)	**0.581**	**(0.521–0.671)**	0.108	(0.090–0.125)
D	OUM	2 + root	0.098	Root	—	—	—	—	**−0.011**	**(−0.044 to 0.017)**	0.114	(0.098–0.124)
				Basal	0.0056 [124]	(0.0011–0.0120)	0.00056	(0.00038–0.00072)	**−0.098**	**(−0.941 to 0.097)**	0.511	(0.263–2.540)
				Pterodactyloidea	0.0056 [124]	(0.0011–0.0120)	0.00056	(0.00038–0.00072)	**1.350**	**(0.836–5.930)**	0.221	(0.104–1.170)
E	OUMA	2	0.067	Basal	**0.0242 [28.6]**	**(0.0197–0.0345)**	0.00165	(0.00118–0.00265)	**−0.008**	**(−0.017 to −0.002)**	0.091	(0.085–0.095)
				Pterodactyloidea	**0.0112 [61.9]**	**(0.0059–0.0203)**	0.00165	(0.00118–0.00265)	**0.939**	**(0.725–1.730)**	0.121	(0.063–0.249)
F	OUM	3 + root	0.067	Root	—	—	—	—	**−0.021**	**(−0.055 to 0.013)**	0.123	(0.101–0.131)
				Basal	0.0108 [64.2]	(0.0055–0.016)	0.00063	(0.00043–0.00078)	**0.024**	**(−0.161 to 0.113)**	0.286	(0.201–0.460)
				Archaeopterodactyloidea	0.0108 [64.2]	(0.0055–0.016)	0.00063	(0.00043–0.00078)	**0.619**	**(0.494–1.100)**	0.248	(0.174–0.430)
				Ornithocheiroidea	0.0108 [64.2]	(0.0055–0.016)	0.00063	(0.00043–0.00078)	**0.910**	**(0.741–1.500)**	0.130	(0.089–0.224)
G	OUMVA	2	0.061	Basal	**0.0352 [19.7]**	**(0.0252–0.0638)**	**0.00112**	(0.00093–0.00173)	**0.006**	**(−0.008 to 0.023)**	0.070	(0.060–0.074)
				Pterodactyloidea	**0.0126 [55.0]**	**(0.0066–0.0210)**	**0.00697**	(0.00298–0.06560)	**1.370**	**(0.972–3.170)**	0.193	(0.117–0.494)

AICc, Akaike’s Information Criterion for finite sample sizes; OU, Ornstein–Uhlenbeck model.

The parameters of models of adult pterosaur body size evolution with non-negligible support are shown. These results are drawn from comparisons among single- and multi-regime generalized OU models^67^. Median parameter values and their absolute ranges are given for analyses conducted on 25 time-calibrated phylogenies. OUM is an OU model with group-specific trait optima (*θ*, in log_10_m); OUMV allows group-specific *θ* and stochastic rate parameters (*β*, Brownian variance in log_10_ (metres)/Ma); OUMA allows a group-specific *θ* and an attraction parameter (*α*). The trait optimum (*θ*) for each group equals the ancestral node value (*Z*_0_), except in models D and F, in which separate *θ* and *Z*_0_ are specified for basal (non-pterodactyloid) pterosaurs. The phylogenetic half-life (ln(2)/*α*) is the time in millions of years (Ma) taken for an OU process to erase half the phylogenetic covariance between sister taxa. Parameter values varying between groups are shown in bold. Only models with ~0.5 times the median AICc weight of the best model, or better, are shown.

**Table 2:** Results of generalized least squares regression of wingspan on other skeletal measurements.

**Explanatory variable**	**Non-phylogenetic**		**Phylogenetic**		***λ*** **estimate**	***N***	**No. of estimates**	**Mean standard estimate error**
	***R***^**2**^	**AICc**	***R***^**2**^	**AICc**				
Skull length	0.82	540.8	0.64	533.7*	0.92	37	35	—
Mandible length	0.87	528.7	0.71	525.2*	0.89	37	35	0.085
Rostrum length	0.71	527.0	0.52	514.9*	0.94	35	29	—
Humerus length	0.94	634.9	0.87	622.1*	0.91	46	27	0.063
Ulna length	0.95	620.7*	0.85	628.3	−0.07	46	19	0.068
Radius length	0.95	624.6*	0.84	630.7	−0.06	46	19	—
Metacarpal IV length	0.87	668.2	0.73	656.3*	0.95	46	11	—
Phalanx IV-1 length	0.97	607.7	0.92	601.1*	1.07	46	16	0.054
Phalanx IV-2 length	0.95	625.5	0.90	611.7*	0.90	46	12	0.039
Phalanx IV-3 length	0.78	689.5	0.82	643.4*	1.08	46	6	—
Phalanx IV-4 length	0.60	504.3	0.85	456.5*	1.11	35	5	—

Mean standard estimate error, the s.d. of differences between wingspans estimated from each regression model, and actual measured wingspans for taxa with known wingspans (only presented for selected, best variables); N, sample size; No. of estimates, the maximum number of additional estimates of unknown wingspans enabled by the explanatory variable.

The coefficient of determination (*R*^2^) and Akaike’s Information Criterion (AICc; the best is indicated with an asterisk*) are given for non-phylogenetic (*λ*=0) and phylogenetic (*λ*=1) regression models. An independent, maximum-likelihood estimate of *λ* (phylogenetic signal strength) is also given (*λ* estimate). Results are shown from one representative time-calibrated tree, and exclude *Jeholopterus*.
